# Who you are or what you have: Historical trends in identity-first and person-first language in books and news media

**DOI:** 10.1371/journal.pmen.0000684

**Published:** 2026-09-16

**Authors:** Jemima Kang, Naomi Baes, Alsa Wu, Nick Haslam

**Affiliations:** 1 Melbourne School of Psychological Sciences, University of Melbourne, Parkville, Victoria, Australia; 2 School of Computing and Information Systems, University of Melbourne, Parkville, Australia; Département des Yvelines / INSERM, FRANCE

## Abstract

Whether people experiencing mental ill health or disability should be referred to using person-first (e.g., “person with autism”) or identity-first (e.g., “autistic person”) terminology is an ongoing subject of debate. The present study conducted the most comprehensive investigation to date of historical trends in the use of the two kinds of terms, evaluating the frequency of “X person/people” relative to “person/people with X” expressions for a collection of mental health, disability, and other conditions in Google Books (1980–2019) and the News on the Web corpus (2010–2025). Consistent with predictions, person-first language became more popular from 1980 onward, but this trend was counteracted by a more recent swing toward identity-first language, resulting in a curvilinear trend. Identity-first language was also more prevalent in singular than in plural expressions. Beyond these broad trends, there was substantial diversity in terminological preferences and trends across conditions. Implications are discussed.

## Introduction

How should we refer to people who are experiencing illness or disability? Driven by the desire to reduce stigma and replace pejorative expressions, advocates have argued for an assortment of terminological changes in recent decades. Deciding on appropriate language has proven to be challenging, however. Debates about the merits of proposed solutions are ongoing.

Arguably the most influential change was proposed by disability activists in the 1980s. Advocating for “person-first” (PF) language [1], they argued that common expressions like “disabled person” or “paraplegic” equated the person with their disability in a way that was reductive and stigmatizing. As an alternative, advocates proposed expressions such as “person with a disability” or “person with paraplegia” that foreground the individual’s personhood and do not define them by what they are unable to do. PF language quickly became recommended practice in academic writing and in media reporting, with guidelines of the US National Rehabilitation Association recommending PF expressions as early as 1985. PF language has been recommended in the American Psychiatric Association’s Diagnostic and Statistical Manuals since 1987 (DSM-III-R) [2] and was recommended in the American Psychological Association’s Publication Manual until the 2020 edition [3].

More recently, in the 2010s, there has been a backlash against PF language, led by disability scholars and psychologists [4,5]. Proponents of “identity-first” (IF) language argued that PF expressions imply that disability is a devalued, unwanted, and peripheral aspect of a person’s identity which can be separated from them. On this view, PF language assumes a medicalized understanding of disability as a bodily affliction that detracts from personhood, implying that it is an undesirable condition that should be minimized or de-emphasized rather than a valued form of human diversity. IF language advocates therefore recommended the use of expressions such as “autistic person” that place the person’s disability front and center.

Although IF language advocacy began in the field of disability, including in relation to neurodevelopmental conditions at the intersection of disability and mental ill health, some writers have argued that it can be appropriate in the psychiatric domain as well. Sass [6], for example, addressed the following question:

Is schizophrenia best conceptualized as a disease entity that comes, as it were, from outside, and with which the sufferer must contend, as with a burden or an enemy? Or is it better seen as a more intimate factor: something that emerges from within, permeates the very core of one’s being, and must, perhaps, be understood as an aspect of the sufferer’s very soul? (p. 395)

Although “person with schizophrenia” had become the preferred expression at the time, he argued, schizophrenia transforms rather than merely qualifies the self. Sass contended that PF language over-simplifies the psychological reality of schizophrenia, implies a biomedical framing that can be stigmatizing, and prevents genuine engagement with ‘schizophrenic’ experience.

Much of the literature on PF and IF language explores the theoretical and political dimensions of the issue, but a growing empirical literature examines the terminological preferences of stakeholders. The evidence to date suggests that these preferences vary as a function of historical time, condition, and personal identification with the condition, but questions whether they have implications for stigma. For example, in early research Lynch et al. [7] found that government employees tended to prefer PF language when referring to disability, but one decade later Bickford [8] found a preference for IF language among blind people. Several recent studies [9–13] have demonstrated broad preferences for IF language among autistic adults with more inconsistent preferences in earlier studies. People with stronger autistic identities had stronger preferences for IF terms [14]. However, studies have found no link between people’s terminological preferences and their desire for social distance from people with a range of mental health conditions [15], and no tendency for people who prefer PF language to show greater tolerance [16] or lower addiction stigma [17].

Empirical studies of the actual use of PF and IF expressions rather than preferences for them have been relatively scarce, and although changes in language use are probable given the history of advocacy efforts, these studies have rarely examined temporal trends. To date, studies have examined language use in academic writing, news media, or organizational websites, and have focused on a small range of conditions; primarily intellectual and other disabilities, obesity, depression, and some medical conditions (e.g., prostate cancer and Parkinson’s disease). Only three studies have explored historical change longitudinally.

La Forge [18] examined all articles published by three rehabilitation journals in 1988, finding that PF language about disability was used in 71% of article titles, a steep rise from the 27% reported in a study of articles from 1984-1985 [19]. Barnish [20] found that PF language was rare overall (19.5%) in academic articles on prostate cancer, celiac disease, depression, and Parkinson’s disease, but almost doubled from 1994-1998 to 2009-2013. Differences between conditions were also apparent: depression was much more likely to be referred to in PF language than Parkinson’s disease (30.7% versus 8.7%). PF language was markedly more prevalent in the mission statements published on disability support group websites (64.0%) than in information for prospective service users published on the websites of family medical centers (15.6%) [20]. Gernsbacher [5] showed that PF language about children with disabilities increased and IF language decreased from 1985 onward in the Google Books corpus. In contrast, rates of PF and IF language about adults with disabilities remained relatively stable over time. These complex, age-moderated patterns also emerged in language about autism. Gernsbacher noted that despite its rising dominance in the disability domain, PF language is used most often to refer to the most stigmatized disabilities and is used to refer to children with disabilities more than those without, implying that it marks rather than erodes stigma.

Researchers from a corpus linguistics background have examined PF and IF language use in finer linguistic detail than the psychology and disability specialists who conducted the previous studies. Halmari [21] extracted references to “mental retardation” from the Houston Chronicle and Google News between 2002 and 2007, classifying phrases as politically correct or incorrect. The former were defined as PF (e.g., “person with mental retardation”) and the latter included typical IF pre-modified expressions (e.g., “mentally retarded man”) as well as nominal adjectival forms (e.g., “the mentally retarded”), predicate adjectival forms (“he was mentally retarded”), and “suffering from” expressions. PF phrases were in the minority in both corpora (26% Houston Chronicle, 27% Google News) and were more commonly used when referring to vulnerable targets such as children and non-criminal adults. This finding revealed “a hypocrisy of sorts according to which some groups are seen as more deserving of the ‘people ﬁrst’ language than others” [21, p. 838].

More recently, Bednarek et al. [22] investigated obesity-related language use in a corpus drawn from 12 Australian newspapers published between 2008 and 2019. They found that contrary to weight-related publication guides, “condition-first” language, which included IF expressions such as “obese person” and “the obese”, was used much more often than PF language. There was no temporal trend in language use over the 11-year study period. PF language was more common in broadsheets than tabloids, suggesting that PF language was aligned with compassion for targets and a progressive political stance, consistent with [21]. That alignment may be specific to particular target groups, however. Bednarek and colleagues write that “support for person-first (people-first) language such as people with obesity is universal” [22, p. 425], but it is far from universal for autism, for example (e.g., Buijsman et al. [9]).

In the most comprehensive study of mental illness-related terminology to date, Price [23] examined PF and IF language in a 1984–2014 UK newspaper-based corpus. PF expressions were much less common in references to agoraphobia, bulimia, anorexia, and schizophrenia than IF expressions, but became more popular in references to mental illness and depression over the study period. Trends in the use of IF language were not reported. Price observed that PF language was especially common when referring to plural “people” rather than singular “person” – often being used in the context of commentary about the stigmatization of mental illness – without formally testing whether PF or IF language use varied by grammatical number in this way. IF expressions about mental illness were frequently stigmatizing in her corpus.

These studies of PF and IF language use demonstrate that it can be assessed rigorously in a range of corpora, that it varies by condition and medium (e.g., academic versus news media), and that it has undergone some historical shifts. However, existing studies have several limitations. First, relatively few target conditions have been examined, and very little research has been conducted in the mental health domain [5,20,23]. Second, many studies have focused on a single target condition (e.g., intellectual disability, disability in general, obesity), preventing systematic comparison between conditions. Third, historical change in language use has rarely been a research focus, and with the exception of Barnish [20] and Price [23], with their 20- and 30-year spans, most have had a relatively short temporal range. As the corpora employed in these two studies ended in 2013 and 2014, respectively, they cannot illuminate trends over the past decade, during which IF language has been increasingly promoted.

The present study aimed to advance the empirical literature on PF and IF language use by addressing these three limitations. Applying a consistent analytic approach in two distinct, very large text corpora to enable ready comparison, we studied a large sample of conditions, most of which were mental health-related. In one corpus we examined “long-run” changes in language use over a 40-year period (1980–2019), the longest span of any study to date, and in the other we examined “short-run” changes over an overlapping 16-year period (2010–2025) that concluded at the time of writing. Rather than track IF and PF expressions separately, we examined their relative prevalence using a simple, intuitive index (i.e., IF expressions as a proportion of all IF and PF expressions for a given year).

The study tested three hypotheses. First, as advocacy for PF language primarily emerged in the 1980s, we predicted a general long-run decline in IF language (relative to PF language) from 1980 to 2019 (H1). Second, as advocacy for IF language rose more recently, we predicted a positive curvilinear trend decelerating or reversing that long-run decline from 1980 to 2019 (H2), a trend that would also be expected to emerge in the short-run analysis (2010–2025). Third, we made the novel prediction that IF language would be more prevalent for singular targets (e.g., “depressed person”) than for plural targets (e.g., “depressed people”) (H3). This prediction was motivated by evidence that individuals are universally perceived to be more real and stable than collectives [24]. Individuals would therefore appear to be more ontologically suitable targets for identity claims than groups or social categories. In addition to our three hypotheses, we explored two exploratory research questions regarding heterogeneity among conditions. We examined whether (a) PF and IF language were generally more prevalent for some conditions than others (RQ1), and (b) whether these conditions differed in their temporal trends (RQ2).

## Method

### Text corpora

The analyses used pre-specified search expressions applied to two large text corpora comprising English-language books and web-based newspapers and magazines, rather than manual selection of individual source documents. The extraction scripts, target-expression list, derived frequency tables, and analysis scripts are available at the following GitHub repository for reproducibility.

#### Google Books.

The long-run analyses drew on the Google Books English 2019 corpus [25], a large diachronic dataset comprising over eight million digitized English-language books published between 1800 and 2019 (totaling 1,997,515,570,677 words). This corpus aggregates material from American, British, Australian, Canadian, and other non-classified English regions, providing a broad sample of published English across two centuries. The corpus supplies yearly normalized frequencies for single words and multi-word expressions, enabling long-term quantitative tracking of language use. Analyses in the present study focused on 1980–2019, corresponding to the approximate beginning of PF language advocacy and the end date of the corpus at the time data were extracted.

Normalized term frequencies were retrieved directly from the Google Books Ngram Viewer JSON API (https://books.google.com/ngrams). Queries were programmed to send HTTPS requests to the API endpoint specifying (i) the list of target expressions, (ii) the corpus ID, and (iii) the time window (1980–2019), with a smoothing parameter of zero to obtain unsmoothed yearly values. Searches used the Google Books English 2019 corpus ID 26 and case-insensitive matching. The extraction initially retrieved frequencies for 1940–2019, after which the reported analyses were restricted to 1980–2019. Because Google Books Ngram provides aggregated yearly frequency data rather than document-level records, no individual books were manually selected or excluded. For each expression, the API returns its yearly relative frequency, defined as the proportion of all tokens in each year that are instances of that expression. These values are proportions of all tokens in the corpus for a given year and can be converted to occurrences per million words by multiplying by 1,000,000.

#### News on the Web.

The short-run analyses drew on the NOW (News on the Web) corpus, which is a daily augmented corpus that contains articles from English-language web-based newspapers and magazines from 2010 to the present [26]. At the time of data collection, the corpus contained approximately 23.6 billion words across sources from 20 countries, including Australia, New Zealand, Malaysia, the United States, Canada, Hong Kong, the United Kingdom, and South Africa. Sources span a broad range of outlets and topic areas; Australian sources for example include ABC, The Age, The Sydney Morning Herald, The Conversation, The Australian Financial Review, Brisbane Times, Yahoo Sports, and 9 News. For each target expression, we counted all case-insensitive matches across the full corpus and aggregated these annually from 2010 through 2025. Counts reflected raw frequency of IF and PF expressions rather than document-level frequency, such that multiple occurrences of an expression within a single article each contributed to the annual total.

### Materials and measures

The study examined the relative prevalence of IF and PF expressions for a set of 15 target conditions in the two distinct text corpora. A relatively broad sample of target conditions was selected that encompassed many of the conditions that have been the focus of the IF/PF language debate. Furthermore, we selected conditions that have a conventional adjectival form and excluded those without one (e.g., ADHD, OCD). The resulting sample predominantly contained diverse mental health and neurodevelopmental conditions (11 of 15), including the generic term mental illness, but also contained non-mental health conditions (diabetes, disability, epilepsy, obesity). The IF and PF terms used to represent the sample of conditions are presented in Table 1.

**Table 1:** Sample of terms representing the 15 target conditions.

Adjectival form (IF)	Noun form (PF)
alcoholic	alcoholism
anorexic	anorexia
anxious	anxiety
autistic	autism
bipolar^a^	bipolar
bulimic	bulimia
depressed	depression
diabetic	diabetes
disabled	disability
dyslexic	dyslexia
epileptic	epilepsy
mentally ill	mental illness
obese	obesity
psychotic	psychosis
schizophrenic	schizophrenia

^a^“Bipolar” appears in both the adjectival and noun columns because the same surface form was used to extract both the IF (e.g., “bipolar person/ people”) and PF (e.g., “person/people with bipolar”) expressions. These expressions were not counted as standalone occurrences of the word “bipolar”; they were counted only when they appeared in the specified IF or PF phrase templates detailed in the 2x2 design, representing the IF/PF and singular/plural conditions.

For each target condition, we computed frequency counts for all combinations of grammatical number (singular vs. plural) and expression type (IF vs. PF). Counts were calculated separately for each year in each corpus. For example, the four different expressions for *anorexia* were “person with anorexia” (singular PF), “people with anorexia” (plural PF), “anorexic person” (singular IF), and “anorexic people” (plural IF). IF and PF language can take a wider variety of forms [21,22]. For example, IF expressions could include plural noun forms such as “anorexics” or “the anorexic” and compounds (e.g., “anorexic and anxious people”), PF expressions could include qualifiers (e.g., “person with severe anorexia”), and both IF and PF expressions could include numerous alternatives to “person” (e.g., “woman with anorexia”, “anorexic Melburnian”). However, our approach captures the most basic and common IF and PF expressions in a way that simplifies the search process, allows for a straightforward 2x2 approach to data analysis, is compatible with the focus of previous studies (e.g., Price [23]), and offers a consistent approach across a diverse collection of conditions. Work by Bednarek et al. [22] shows that allowing qualifiers or other intervening terms only marginally increased the detected frequency of relevant expressions, and allowing over 100 nouns other than “person” or “people” substantially increased the frequency of IF but not PF expressions. Therefore, although our analysis omits a proportion of IF and PF expressions in a way that could alter some findings, we believe this omission is not likely to be critical on balance.

For each target condition, year, and grammatical number (singular vs. plural) in each corpus, we computed an index of the prevalence of IF language relative to PF language. The index was defined as the frequency of IF expressions divided by the combined frequency of IF and PF expressions. For example, for singular expressions involving *schizophrenia*, we divided the frequency of “schizophrenic person” by the summed frequency of “schizophrenic person” and “person with schizophrenia.” The index ranges from 0 (entirely PF expressions) to 1 (entirely IF expressions), with 0.5 representing equal prevalence of PF and IF expressions. As a simple proportion, the index is not affected by variation in the number of expressions per year. Cases with no IF or PF expressions in a given year were treated as missing.

### Statistical analysis

For both the long run and short run analyses, yearly variation in relative use of IF language was modeled using a linear mixed-effects model. The dependent variable was the yearly IF index for each target condition. Three fixed effects were included in the long-run analysis: (1) a linear effect of year (z-scored) to test for an overall historical decline in IF language (H1); (2) a quadratic effect of standardized year to test for the predicted curvilinear trend (long-run analyses only; H2); and (3) grammatical number (plural vs. singular) to test whether IF usage is systematically higher for singular expressions than for plural ones (H3). As the short-run analysis involved a relatively brief time series and there was no expectation of a curvilinear effect, only fixed effects (1) and (3) were included in it. Grammatical number was included as a fixed effect in both analyses because the theoretical prediction underlying H3 concerns a baseline difference between singular and plural expressions, not separate temporal trajectories. Thus, the model estimates a single historical trend applied to both forms (plural and singular).

To address the two exploratory research questions (RQs), whether conditions differed in their prevalence level (RQ1) and temporal trajectories (RQ2) of IF language, the model included condition-specific random effects. Random intercepts were included to allow conditions to differ in their baseline levels of IF language (RQ1) and random slopes for year were added to assess whether conditions followed distinct temporal patterns (RQ2). In the long-run analysis, condition-specific random slopes were specified for both the linear and quadratic year terms. In the short-run analysis, random slopes were included for the linear year term only. All predictors were included simultaneously, and the model was fitted using restricted maximum likelihood (REML). Confidence intervals for fixed-effect estimates were Wald-type 95% intervals calculated from the fitted mixed-effects model using the model-estimated standard errors.

## Results

### Long-Run Analyses (1980–2019)

The intraclass correlation coefficient (ICC), estimated from an intercept-only mixed-effects model, indicated substantial clustering by condition (ICC = .775), supporting the use of a mixed-effects approach. Between-condition differences accounted for ~78% of the variance in IF language in the Google Books corpus. The linear mixed-effects model revealed clear support for all hypotheses (see Table 2). Consistent with H1, the fixed effect of year (z-scored) was significantly negative (b=−0.09, 95% CI [−0.12,−0.06], *p* < .001), indicating that IF proportion decreased by approximately 0.09 (9 percentage points) for each one-standard-deviation increase in year across 1980–2019. Supporting H2, the quadratic effect of year was significantly positive (*b* = 0.032, 95% CI [0.014, 0.051], p = .001), indicating a deceleration of the downward trajectory in IF use over time, although this did not produce a reversal of the direction of the trend. Strong support was also found for H3: IF usage was significantly higher for singular expressions than for plural expressions, as evidenced by the positive effect of grammatical number (*b* = 0.172, 95% CI [0.162, 0.183], *p* < .001), corresponding to an average difference of approximately 0.17 on the index. This indicates that in the Google Books corpus, IF expressions were employed substantially more to refer to individuals (e.g., “depressed/autistic person”) than to groups (e.g., “depressed/autistic people”).

**Table 2:** Fixed effects from the long-run mixed-effects model predicting yearly identity-first proportion from year (z-scored; linear and quadratic terms) and grammatical number (singular versus plural).

Predictor	Estimate	SE	95% CI	z
Intercept	0.387***	0.086	[0.22, 0.56]	4.50
Number [singular]	0.172***	0.005	[0.16, 0.18]	32.25
Year	-0.088***	0.016	[-0.12, -0.06]	-5.37
Year^2^	0.032***	0.010	[0.01, 0.05]	3.40

*** p < .001. Confidence intervals are Wald-type 95% intervals generated from the fitted mixed-effects model using model-estimated standard errors.

**Fig 1:**
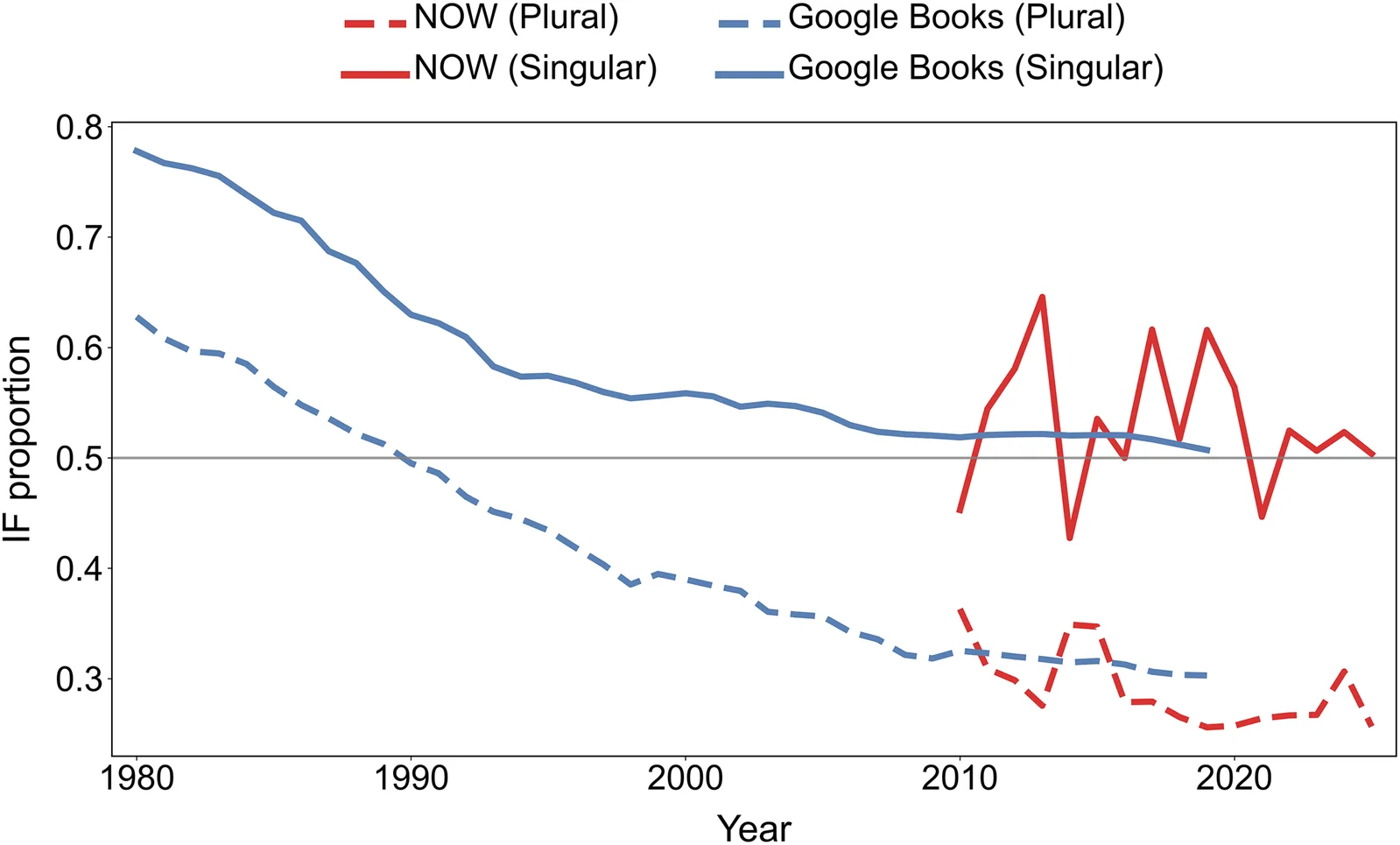
Average trends in the IF index across the 15 conditions for singular and plural expressions for the long-run and short-run analyses. Solid lines = singular expressions, dashed lines = plural expressions, Google Books corpus = blue lines, NOW corpus = red lines.

Fig 1 (blue lines) presents the average trend across the 15 conditions. The proportion of IF terms declines substantially relative to PF expressions from 1980 to 2019 for both plural and singular forms. For plural forms, the IF proportion decreases from approximately 0.62 in 1980 to 0.32 in 2019, indicating that PF expressions predominated later in that period. Singular forms start from a higher baseline (0.81 in 1980) and follow a similar downward trajectory, reaching about 0.51 by 2019, indicating approximately equal prevalence of IF and PF expressions. The positive quadratic effect flattens or stabilizes the trends in later years. Fig 1 indicates that the decline in IF language began to slow appreciably in the early 1990s for singular expressions and more gently for plural expressions in the late 2000s. Noisiness in the short-run trends rules out interpretation of any discontinuities in them.

**Fig 2:**
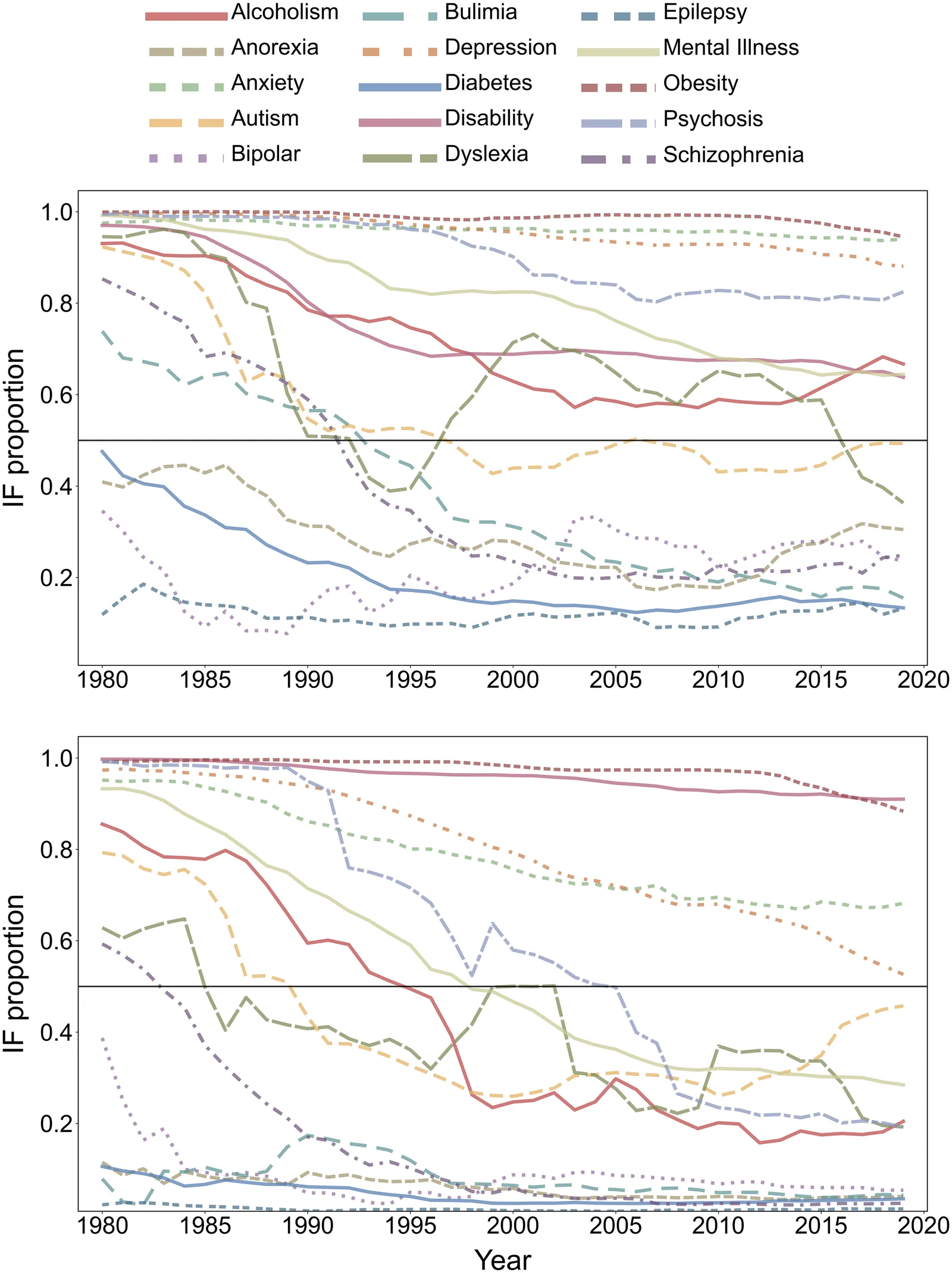
Long-run IF proportions for each of the 15 conditions for singular and plural expressions. Top panel = singular expressions, bottom panel = plural expressions. Horizontal grey line at 0.5 on y-axis indicates equal prevalence of IF and PF expressions.

The random-effects structure of the analysis addressed the two exploratory research questions concerning heterogeneity across conditions. Fig 2 presents the trends for singular and plural expressions for each of the 15 conditions. The random-intercept standard deviation was 0.33 (variance = 0.11), indicating meaningful between-condition differences in baseline IF usage at the centered time point, after accounting for grammatical number and temporal trends (RQ1). In addition, random slopes for the linear (variance = 0.004) and quadratic (variance = 0.001) year terms indicate that diagnostic labels followed divergent long-run trajectories (RQ2). Some conditions showed steep historical declines in IF usage (e.g., *psychotic*, *schizophrenic*, *mentally ill*), whereas others showed gentler declines (*anorexic*, *diabetic*) or relatively stable trajectories (e.g., *bipolar*, *epileptic*). Curvature also varied across conditions: terms such as *autistic* and *schizophrenic* showed relatively strong U-shaped patterns, whereas others (e.g., *depressed*, *obese*) continued to decline steadily. Model-derived linear and quadratic slopes for each condition are presented in Table 3. Together, these findings indicate substantial heterogeneity across conditions in both baseline IF prevalence and temporal change, with Fig 2 highlighting especially pronounced differences in long-run trajectories (RQ2).

**Table 3:** Model-derived condition-specific linear and quadratic time coefficients from the long-run mixed-effects model.

Condition	Linear Slope	Quadratic Slope
bipolar	0.001	0.026
epileptic	-0.005	0.013
obese	-0.019	-0.010
anorexic	-0.039	0.029
diabetic	-0.050	0.036
anxious	-0.056	0.009
disabled	-0.064	0.022
depressed	-0.086	-0.006
bulimic	-0.100	0.019
dyslexic	-0.101	0.038
autistic	-0.119	0.103
mentally ill	-0.164	0.032
alcoholic	-0.167	0.064
schizophrenic	-0.168	0.100
psychotic	-0.180	0.009

Year was standardized (z-scored), so linear and quadratic coefficients are expressed on the standardized year scale. Coefficients combine the population-average fixed effect with condition-specific random effects (best linear unbiased predictions). Conditions are ordered from highest to lowest linear coefficient.

### Short-run analyses (2010–2025)

Equivalent analyses were carried out for the short-run analysis on the NOW corpus data, but without examining the curvilinear effects due to the shorter time period. Additionally, the substantially lower frequency of PF and IF expressions in the corpus reduces the statistical power to detect robust trends for individual conditions. The averaged trend across the 15 conditions is presented in Fig 1 (red lines). The intraclass correlation coefficient (ICC) confirmed the appropriateness of using a mixed-effects analytical approach. Between condition differences accounted for 58% of the variance in IF language for the NOW corpus.

Table 4 reports that the fixed effect of year was not statistically significant (b=−0.012, 95% CI [−0.046, 0.022], *p* = .485). This indicates that there was no overall linear trend in the prevalence of IF language relative to PF language over the 2010–2025 period across the 15 conditions. As shown in Fig 1 (red lines), IF expressions remain relatively stable during the time period. Although this pattern contrasts with the significant negative trend obtained in the long-run analysis (1980–2019), it is consistent with the stabilized trends in later years. Consistent with H3, however, singular expressions showed significantly higher levels of IF language than plural expressions (*b* = 0.241, 95% CI [0.204, 0.279]), *p* < .001, with a mean difference of approximately 0.24. Consistent with the long-run analyses, these results suggest that IF language is used more when referring to individuals rather than groups, which can be seen in Fig 1.

**Table 4:** Fixed effects from the short-run mixed-effects model predicting yearly identity-first proportion from year and grammatical number (singular versus plural).

Predictor	Estimate	SE	95% CI	z
Intercept	0.530***	0.067	[0.399, 0.661]	7.93
Number [singular]	0.241***	0.019	[0.204, 0.279]	12.59
Year	-0.012	0.017	[-0.046, 0.022]	-0.70

*** p < .001. Confidence intervals are Wald-type 95% intervals calculated from the fitted mixed-effects model using model-estimated standard errors.

The random intercept variance revealed meaningful heterogeneity in baseline levels of IF usage across conditions (Var = 0.064). Thus, related to RQ1, the results suggest that there are condition-specific differences in baseline IF usage. Relevant to RQ2, random slopes for year (Var = 0.003) revealed modest variation across conditions, so, although the average trend was not significant, individual conditions showed divergent patterns. Table 5 reports slopes for each diagnostic label in the short-run analysis, with negative values indicating declines in the relative prevalence of IF language over time. Some conditions demonstrated steep declines in IF usage over time (e.g., obesity, depression and anorexia) whereas others showed steep increases (e.g., alcoholism, autism and dyslexia). Together, these findings suggest that there was between-condition variability in both historical trajectories and baseline prevalence.

**Table 5:** Model-derived condition-specific linear time coefficients from the short-run mixed-effects model.

Target	Linear Slope
alcoholic	+0.066
autistic	+0.055
dyslexic	+0.038
bulimic	+0.008
diabetic	+0.011
epileptic	+0.008
schizophrenic	+0.002
mentally ill	-0.014
disabled	-0.019
psychotic	-0.029
anxious	-0.029
bipolar	-0.048
depressed	-0.059
anorexic	-0.069
obese	-0.102

Year was standardized (z-scored). Coefficients combine the population-average fixed effect with condition-specific random effects (best linear unbiased predictions). Conditions are ordered by the linear coefficient.

### Combined trends

The long-run and short-run analyses show several interesting convergences. First, as Fig 1 shows, the short-run NOW corpus trends continue those of the long-run Google Books trends both in level and in trajectory. Second, differences between conditions in short-run linear trends (Table 5) parallel differences between them in long-run curvilinear trends (Table 3). That is, conditions which showed a steeper short-run rise in IF language in the NOW corpus also tended to show a more positive quadratic trend (i.e., upswing in later years) in the Google Books corpus (Spearman’s *r* = 0.70, *p* = .004). The long-run and short-run analyses therefore both support the existence of a relatively recent increase in the use of IF language that is more pronounced for some conditions than others.

**Fig 3:**
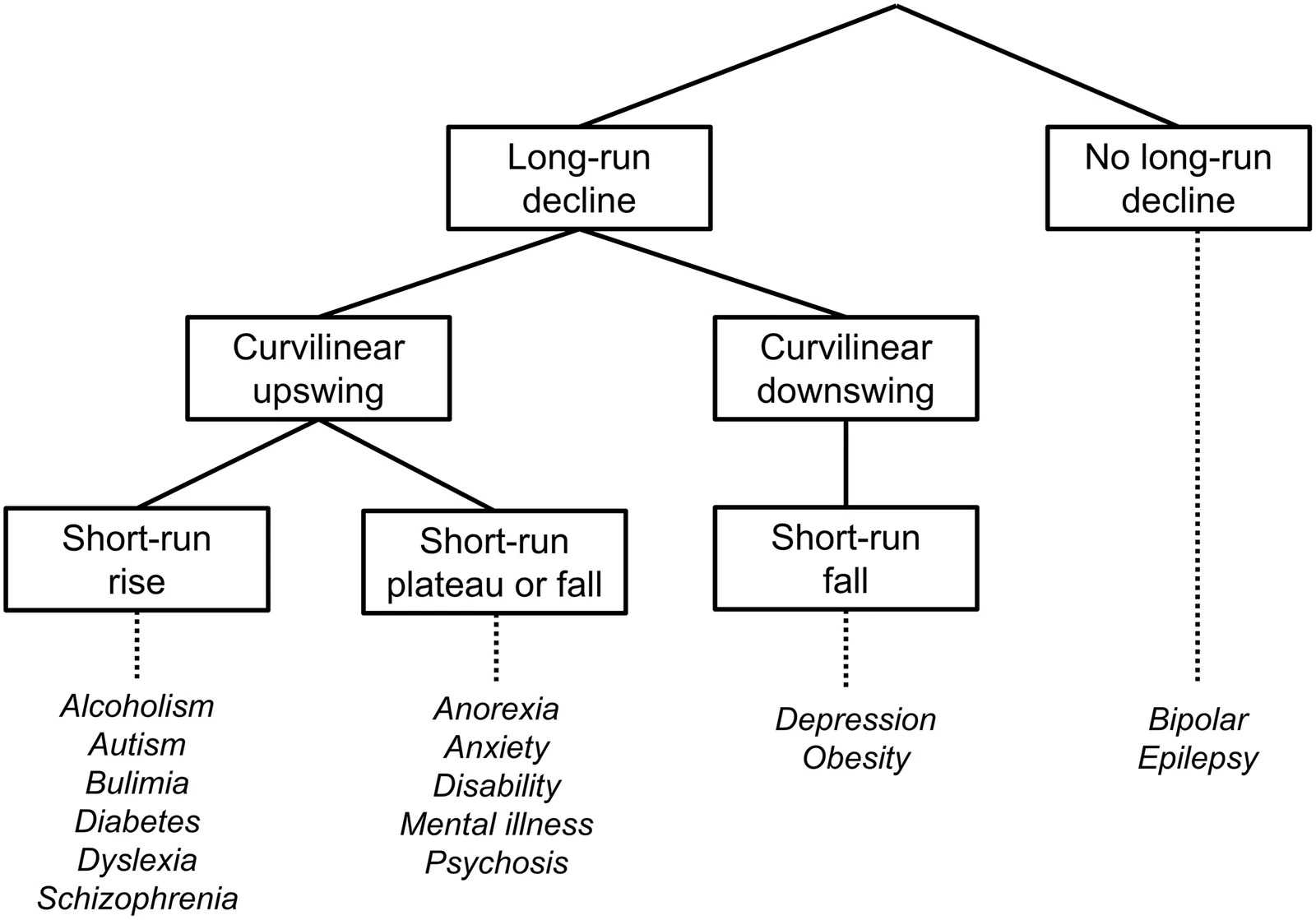
Combined trends for both the short-run linear slopes and long-run curvilinear slopes summarized into four broad groups.

The divergent temporal trends for the 15 conditions can be tentatively assigned to four broad groups (Fig 3). Six of the conditions (*alcoholism*, *autism*, *bulimia*, *diabetes*, *dyslexia*, *schizophrenia*) show a long-run (linear) decline in IF language and a positive long-run (curvilinear) effect with a short-run trend that reverses that decline in recent years. Five show the same linear and curvilinear long-run effects but have a short-run plateau or fall (*anorexia*, *anxiety*, *disability*, *mental illness*, *psychosis*). *Depression* and *obesity* make up a third group characterized by continued long-run declines in IF language, slight negative curvature, and falling short-run trends. The linear decline was close to the overall average for *depression* but relatively weak for *obesity*. Both conditions show a persistently high predominance of IF language throughout the study period. Finally, *bipolar* and *epilepsy* show essentially no long-run decline in IF language. Their short-run slopes differ in direction, so this group is defined primarily by the relative stability of their long-run trajectories. Both of these conditions display a stubbornly high predominance of PF language.

## Discussion

Our findings offer strong support for our hypotheses, demonstrating clear but complex historical trends in the relative prevalence of IF and PF language. The long-run analyses, carried out in a corpus of English-language books, show a substantial decline in preference for IF language from 1980 onward across a diverse range of conditions. However, these analyses also revealed a curvilinear trend, the decline in IF language slowing or beginning to reverse in recent decades. The short-run analysis, conducted in a corpus of English-language news articles from 2010 to 2025, was entirely compatible with the long-run trajectory, showing a relatively flat average trend over this period rather than the decline in preference for IF language revealed by the early decades of the long-run analysis.

These historical patterns are likely to reflect changes in how authors, editorial guidelines, and academic communities frame mental health- and disability-related conditions in response to advocacy and scholarship. The clear decline in IF expressions from the 1980s onwards reflects the growth of advocacy for PF alternatives in clinical, educational, and editorial guidelines during this period. The more recent rebound of IF language is likely to represent a common reframing of mental health and disability under the influence of more recent disability- and neurodiversity-oriented scholarship [27]. How much this rebound can be attributed to specific historical events such as shifts in editorial guidelines is hard to establish in this study. These shifts have been very recent – the American Psychological Association only endorsed IF terminology in 2020 [3] – and could therefore only be detected in principle in the short-run analysis. As that analysis did not reveal an aggregate increase in IF terminology from 2010-2025 or a clear upswing around 2020, it is unlikely to be sufficiently sensitive to detect any such shifts. Further work, perhaps examining academic text rather than news media, is required to time-lock changes in IF language to specific guidelines. However, the fact that the long-run decline in IF language began to slow down in the 1990s suggests that factors other than editorial guidelines have influenced the popularity of IF terms.

In addition to these broad trends, our findings demonstrate substantial heterogeneity among the trends for specific conditions. Most conditions showed a long-run decline in IF language, although its magnitude varied substantially. The decline was especially steep for some conditions (i.e., *alcoholic*, *autistic*, *mentally ill*, *dyslexic*, *psychotic*) and essentially absent for others (i.e., *obese* and *bipolar*). Some conditions increased their relative prevalence of IF language later in the long-run study period (i.e., *autistic*, *alcoholic*, *dyslexic*, *schizophrenic*), whereas others showed no reversal of their downward trend or even accelerated it (i.e., *depressed* and *obese*). Some of these differences among conditions may reflect the main targets of advocacy work, which earlier in the study period focused on promoting a destigmatizing shift towards PF language for disability and severe mental illness, and later focused on an identity-affirming shift toward IF language for neurodivergent and disability-related conditions. Weaker temporal trends would be expected for conditions that have been marginal to most advocacy work, such as eating, mood, and neurological disorders. For example, *bipolar* and *epilepsy* may have maintained a consistently strong preference for PF language because neither has been promoted as a major basis for identity. Alternatively, some term preferences may be more temporally stable for primarily linguistic reasons. “Depressed person” may have remained much more popular than “person with depression” because the adjectival form is familiar and fluent, whereas “person with bipolar” may have remained more popular than “bipolar person” because this clinically similar condition never developed a strongly conventionalized adjectival person-label in the same way as “depressed person”.

Differences in trajectories of preferences for IF versus PF language may also reflect differing beliefs about the fundamental nature of the conditions. PF language has been celebrated and criticized for implying that the condition in question is separable from the person who experiences it. That view is likely to be most credible when the condition is seen as externally caused, transient, and incidental to identity. For example, the consistently low level of IF terminology for *epilepsy* may reflect the judgment that the condition is a random affliction. Similarly, IF language has been celebrated and criticized for implying that the condition in question is deeply embedded in the person and should be most plausible if the condition is viewed as manifesting the person’s unchanging nature. The consistently high preference for IF language for *obesity* may reflect and communicate the view that the condition is intrinsic to and revealing of the person. Viewed in this light, IF language may tend to be employed with conditions that are conceptualized in stable, essentialized ways [28,29], and the rising use of IF terminology for particular conditions may promote more essentialist understandings of them. These understandings can be a double-edged sword: they present the condition as a meaningful basis for identity, but they can also induce pessimism about recovery [30].

Recent work by Guley and colleagues [31] accords with these ideas, finding that people judge some mental health conditions to be more a part of the person’s “true self” than others. Those judged to be biologically caused and with recovery unlikely, such as autism and ADHD, were seen as more part of the true self and those judged to be psychologically caused and transient, such as depression and anorexia, were seen as less so. Autism may be especially likely to attract IF language because it is seen as a true, integral part of the person, and the rise in IF language use in relation to autism may reflect and reinforce increases in its perceived centrality to personal identity. Similarly, the swing back to IF language we observed for schizophrenia may reflect a growing recognition of the condition’s identity-relevance [6].

In addition to the historical trends discussed above, we found strong evidence for our prediction that IF language is more common for singular than plural expressions. This difference was consistent across conditions and over time. Although we believe the effect is novel, albeit foreshadowed by Price [23], it is intuitively plausible. If mental health or disability conditions are understood as attributes of individuals, then using those conditions to define aspects of identity is more appropriate or salient for individuals than for collectives. IF language has been justified by the argument that such conditions are inseparable from the individual, and it should therefore be especially preferred when referring to individuals. However, to the extent that a condition comes to be seen as a collective rather than merely personal identity – a source of group belonging – we might expect the greater use of IF language when referring to individuals to become less pronounced.

The preference for IF language when applied to individuals may have implications for studies of preferred terminology among people with lived experience of the conditions. Numerous studies have been carried out on “autism,” with a recent review finding no clear preference for IF or PF language [32]. These studies typically ask about preferences for singular expressions (“person with X” versus “X person”), but they might profitably also ask about plural expressions: how people think groups of people should be described. Studying possible discrepancies between the singular and plural cases might clarify the complexities of identity surrounding mental health and disability conditions.

This study has several limitations. First, we focused our analysis on simple PF and IF terminology (e.g., “person with depression” and “depressed person”). Previous research has documented a wide range of additional terms specific to particular conditions, such as “person with ASD” or “Aspie” for autism [32], as well as a range of alternative grammatical forms of PF and IF language (e.g., “person suffering from depression” [PF] and “schizophrenic” as a noun [IF]). We chose to examine simpler expressions to enable direct and standardized comparison across a diverse set of conditions. However, future work could incorporate a broader set of terminological variations. Second, some conditions had limited instances in particular years, particularly in the NOW corpus, thereby limiting the capacity to evaluate short-run trends. Although the NOW corpus is very large, future research could address this by incorporating additional news sources to increase coverage. In addition, PF and IF language on social media platforms (e.g., Reddit or X) could be examined, given that considerable disability activism has occurred within online communities [33]. Finally, the present study’s interpretation of differences between conditions in PF versus IF language trends was entirely post hoc, and future studies should test theory-guided predictions about these trends and the factors that influence them.

## Conclusion

Language use in relation to mental health and disability represents what has been a contentious topic for decades. Although a large body of work has examined IF and PF language, our study provides the most comprehensive analysis to date. We show how language use has evolved in response to scholarship, advocacy, and the wider culture. Future work should continue to monitor these trends as this will clarify the broader societal beliefs they reflect and reinforce and ultimately, help to inform the ongoing debate about appropriate ways to refer to individuals experiencing a mental health condition or disability.
